# Pearled papules over tattoo: Molluscum cotagiosum

**DOI:** 10.11604/pamj.2013.16.49.3442

**Published:** 2013-10-11

**Authors:** Ricardo Ruiz-Villaverde, Daniel Sánchez-Cano

**Affiliations:** 1 Dermatology Unit. Complejo Hospitalario de Jaen, Jaen, Spain; 2 Internal Medicine. Hospital Santa Ana, Motril, Granada, Spain

**Keywords:** Molluscum cotagiosum, tattoo, pearled papules, immunocompetent

## Image in medicine

A 23-year-old immune-competent man attended to our dermatology unit complaining asymptomatic pearled papules over a previous black tattoo performed over his right abdominal flank. Past medical history was unremarkable. Physical examination showed several umbilicated skin-coloured papules on the tattooed skin and the surrounding skin. Serology results for syphilis, hepatitis B and C, and HIV were negative. Histology of one of the papules demonstrated multiple molluscum bodies. Curettage in programmed sessions was effective. No recurrences have been observed after 6 months. Molluscum contagiosum (MC) is a viral infection caused by a poxvirus. They are most commonly observed in children with atopic dermatitis although its incidence has increased in adults, predominantly due to HIV infection. Concretly when MC lesions affect perineal area, a sexually transmitted disease (STD) may be suspected. Common STD should be rule out. In our patient the appearance of molluscum lesions on the tattoo may be due to transmission of the virus through the instruments used in its implementation or prior contamination of the ink. Koebner phenomenon or “isomorphic response” is the main cause of the spread that many patients present at the time of consultation. Therapeutical approach includes three categories: a) Destructive: curettage, cryotherapy and topical application of keratolytic agents (potassium hydroxide 5-10% in aqueous solution); b) immunomodulatory: imiquimod 5% cream and c) antiviral: podofilotoxin solution, cidofovir. Otherwise in certain cases papules resolve spontaneously.

**Figure 1:**
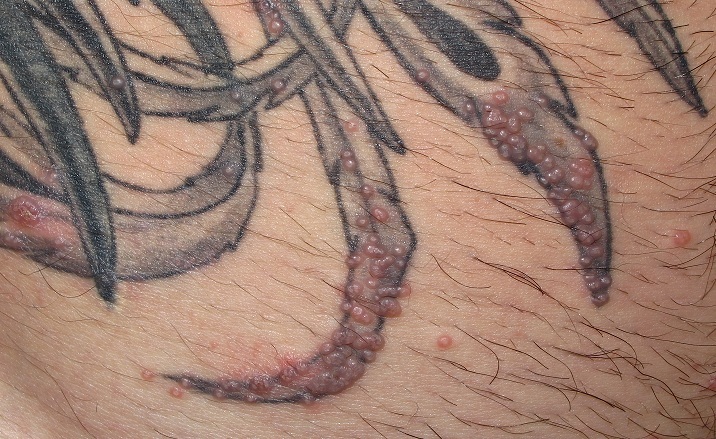
Physical examination showed several umbilicated skin-coloured papules on the tattooed skin and the surrounding skin

